# Genistein in polycystic ovary syndrome: mechanisms, preclinical evidence, and translational potential

**DOI:** 10.3389/fgwh.2026.1836617

**Published:** 2026-07-17

**Authors:** Jialai Wang, Ying Yang, Ziwei Wang, Fengjuan Li, Zhenyu Shi, Tianhang Gao, Hui Chang

**Affiliations:** 1 The First Clinical Medical College, Heilongjiang University of Chinese Medicine, Harbin, China; 2 Department of Obstetrics and Gynecology, First Affiliated Hospital of Heilongjiang University of Traditional Chinese Medicine, Harbin, China

**Keywords:** anti-inflammatory, follicular development, genistein, glucose and lipid metabolism, insulin resistance, oxidative stress, PCOS

## Abstract

Polycystic ovary syndrome (PCOS) is a prevalent reproductive-endocrine disorder in reproductive-aged women. Due to the lack of radical cure, multi-target therapeutic strategies are urgently needed. Genistein, a soy-derived phytoestrogen, exhibits pleiotropic biological activities and is a promising adjuvant candidate for PCOS. Accumulating studies suggests that genistein may improves insulin sensitivity, regulates reproductive hormones, promotes follicular development, and alleviates oxidative stress and chronic inflammation, thereby ameliorating PCOS symptoms. This review summarizes the therapeutic effects, molecular mechanisms, preclinical evidence, and clinical translation prospects of genistein in PCOS, providing a theoretical basis for its clinical application. However, significant challenges remain in translating this research into clinical practice. These include the limited availability of data from human clinical trials, the lack of a clearly defined optimal dosage and administration regimen, insufficient evidence regarding long-term safety, and the potential for variations in bioavailability and individual metabolism to impact efficacy. Addressing these limitations is critical for advancing genistein toward future clinical application.

## 1 Introduction

Polycystic ovary syndrome (PCOS) is the most common heterogeneous endocrine disorder in women of reproductive age, characterized by hyperandrogenism, ovulatory dysfunction, and polycystic ovarian morphology, with a global prevalence of approximately 11%–13% (1). Although the pathophysiology of PCOS is not fully elucidated, it involves genetic and epigenetic susceptibility, dysregulation of the hypothalamic-pituitary-ovarian (HPO) axis, androgen excess, and insulin resistance (IR) (2). Specifically, HPO axis dysfunction elevates the LH/FSH ratio, while compensatory hyperinsulinemia secondary to IR further stimulates androgen production from the ovaries and adrenal glands, which not only exacerbates hyperandrogenism but also worsens IR itself and impairs follicular development, forming a self-reinforcing cycle (3, 4). Clinically, PCOS presents not only with anovulation, infertility, and menstrual irregularities but also confers elevated long-term risks of type 2 diabetes, cardiovascular disease, and psychological disorders (5).

According to the 2003 Rotterdam criteria, PCOS is diagnosed when at least two of the following three features are present after exclusion of other causes: clinical or biochemical hyperandrogenism; ovulatory dysfunction, including oligo- or anovulation; and polycystic ovarian morphology on ultrasound. Common phenotypes include phenotype A (all three features), phenotype B (hyperandrogenism and ovulatory dysfunction), phenotype C (hyperandrogenism and polycystic ovaries), and phenotype D (ovulatory dysfunction and polycystic ovaries without hyperandrogenism) (6). Based on clinical and mechanistic profiles, PCOS can also be divided into four proposed subtypes: a hyperandrogenic subtype, an obese/metabolic subtype, a high SHBG subtype, and a high LH/AMH subtype (7).

Current therapeutic strategies for PCOS primarily comprise lifestyle intervention, insulin sensitizers, hormonal therapy, and ovulation induction agents. However, these modalities are frequently limited by variable efficacy across individuals, adverse effects, suboptimal patient adherence, and a paucity of long-term safety data (8). Lifestyle interventions, focusing on diet and physical activity to control weight and prevent complications, form the cornerstone of management. Indeed, evidence suggests that comprehensive lifestyle modifications, particularly when combined with weight reduction, can yield sustained improvements in metabolic and hormonal parameters (9). Similarly, insulin-sensitizing agents like metformin have demonstrated efficacy in improving hyperinsulinemia, hyperandrogenism, and ovulatory function, as corroborated by recent meta-analyses (10, 11). Drug therapy includes combined oral contraceptives to regulate menstrual cycles and control hirsutism, anti-androgens such as spironolactone for resistant symptoms, and ovulation-inducing agents such as letrozole and clomiphene for infertility. However, these interventions may not fully modify the long-term progression of the syndrome (12) and may cause adverse effects, including gastrointestinal symptoms with metformin, symptom recurrence after stopping oral contraceptives (13), menstrual irregularity and teratogenic risk with spironolactone, and increased risk of miscarriage or multiple pregnancy with ovulation induction agents (14). Therefore, novel multi-target therapies that are effective and well tolerated are still needed.

In this context, genistein, a soybean-derived isoflavone with phytoestrogenic properties, has emerged as a prominent focus for potential adjunctive PCOS therapy due to its combined antioxidant, anti-inflammatory, and pro-autophagic activities. These properties have demonstrated clear clinical benefits in non-PCOS age-related conditions, including hypertension, metabolic disorders, Alzheimer's disease, and osteoporosis (15). Given that the core pathophysiology of PCOS likewise involves metabolic dysregulation, chronic inflammation, and oxidative stress imbalance, genistein is hypothesized to hold translational potential for PCOS intervention. Current preclinical evidence suggests it may improve insulin sensitivity and regulate glucose and lipid metabolism. It may also support sex hormone balance, follicular development, and ovarian function while reducing chronic inflammation and oxidative stress (16). However, clinical translational evidence in this domain remains scarce, underscoring the urgent need for rigorous validation in PCOS-specific cohorts.

This narrative review summarizes the recent advances regarding genistein in the management of PCOS, with emphasis on its regulatory effects on insulin resistance, lipid metabolism, granulosa cell function, follicular development, inflammation, and oxidative stress. Furthermore, it analyzes the current limitations in existing research and proposes future translational strategies, with the aim of clarifying its clinical therapeutic potential and bridging the gap between preclinical evidence and clinical application.

## 2 Structural characteristics and pharmacological activities of genistein

**Figure 1:**
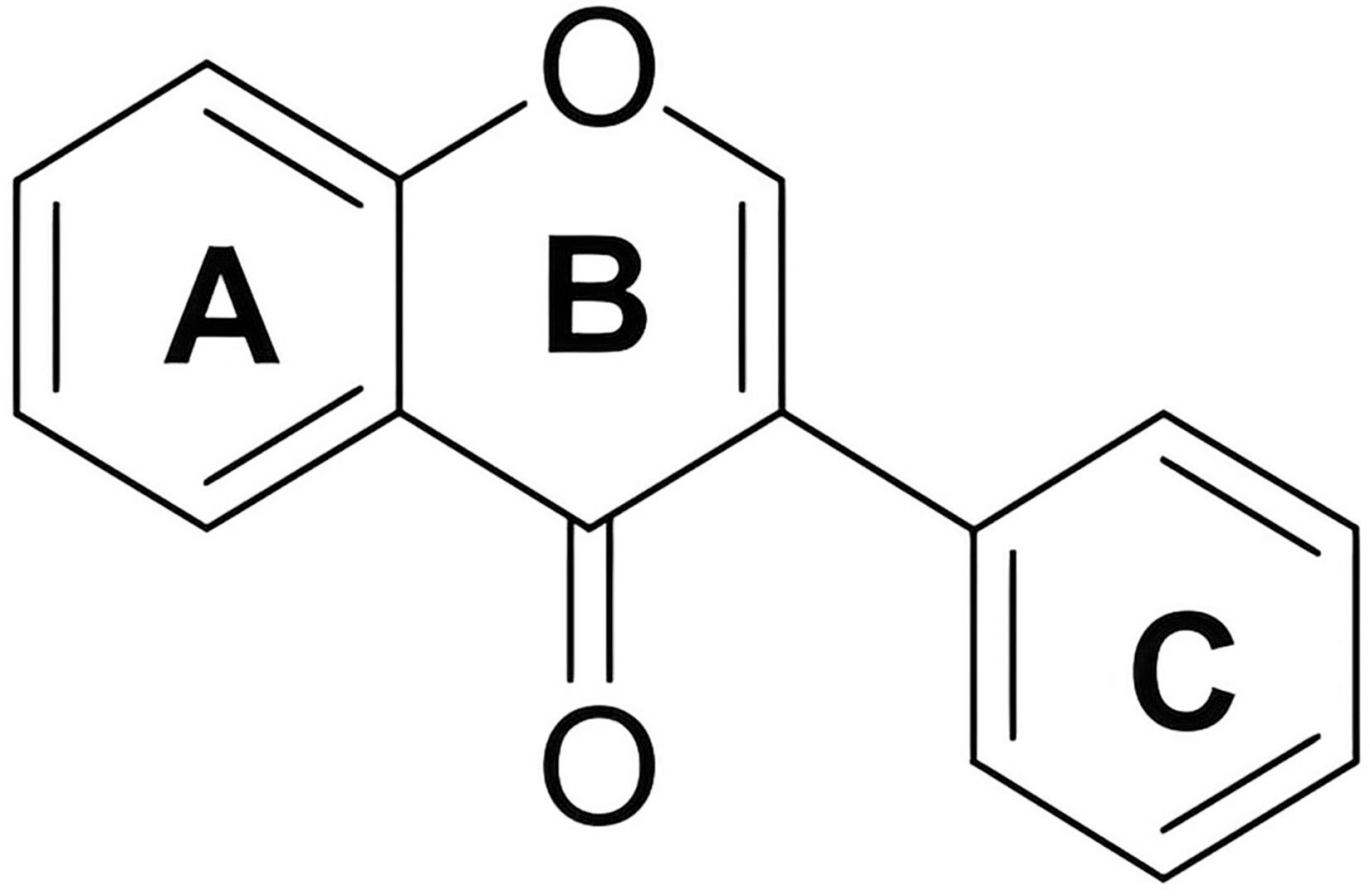
Genistein: chemical structure of 3-phenylchromen-4-one. Adapted from (101).

Genistein (4′,5,7-trihydroxyisoflavone) exists in both aglycone and glycoside forms, with the glycoside form serving as the main transport and metabolic form *in vivo* (17). Its molecular structure contains two aromatic rings (A and B) and an oxygen-containing heterocycle (C ring), forming the typical 3-phenylchromen-4-one skeleton (Figure 1). This rigid planar structure contributes to its chemical stability and underlies its biological activity (16). The phenolic hydroxyl groups in genistein resemble estradiol, allowing it to bind estrogen receptor subtypes with bidirectional modulatory effects. It can act as either an agonist or an antagonist depending on the physiological context (18, 19). In addition, the 4′,5,7-trihydroxy structure gives genistein strong antioxidant activity, and genistein-derived carbon dot materials have also shown anti-inflammatory properties (20, 21). This structure-dependent multi-target profile may contribute to the therapeutic potential of genistein, which has been reported in metabolic syndrome, diabetes, cardiovascular disease, osteoporosis, and cancer (22, 23).

## 3 Therapeutic effects of genistein on metabolic dysfunction in PCOS

### 3.1 Genistein improves insulin resistance

Insulin resistance (IR) is a pivotal driver in PCOS (24), affecting approximately 75%. Compensatory hyperinsulinemia exacerbates hyperandrogenism by stimulating ovarian and adrenal androgen production, disrupts HPO axis function, and impairs β-cell function (25). In addition, IR is an independent risk factor for early miscarriage and adverse pregnancy outcomes in women with PCOS (26). Furthermore, as a central mechanism underlying hyperglycemia, dyslipidemia, and hypertension, IR also increases cardiovascular risk in this population (27).

At the molecular level, IR is closely associated with disruption of the glucose transporter type 4 (GLUT4)-mediated glucose uptake pathway (28). Under normal conditions, insulin activates the insulin receptor substrate (IRS)/phosphoinositide 3-kinase (PI3K)/protein kinase B (Akt) signaling pathway (29). This promotes GLUT4 translocation from intracellular stores to the plasma membrane, allowing glucose uptake in muscle and adipose tissue and maintaining glycemic balance (30–32). However, in the state of IR, this signaling pathway is disrupted at an early stage. As a result, GLUT4 translocation is impaired, glucose uptake decreases, and hyperglycemia with compensatory hyperinsulinemia develops (33).

**Figure 2:**
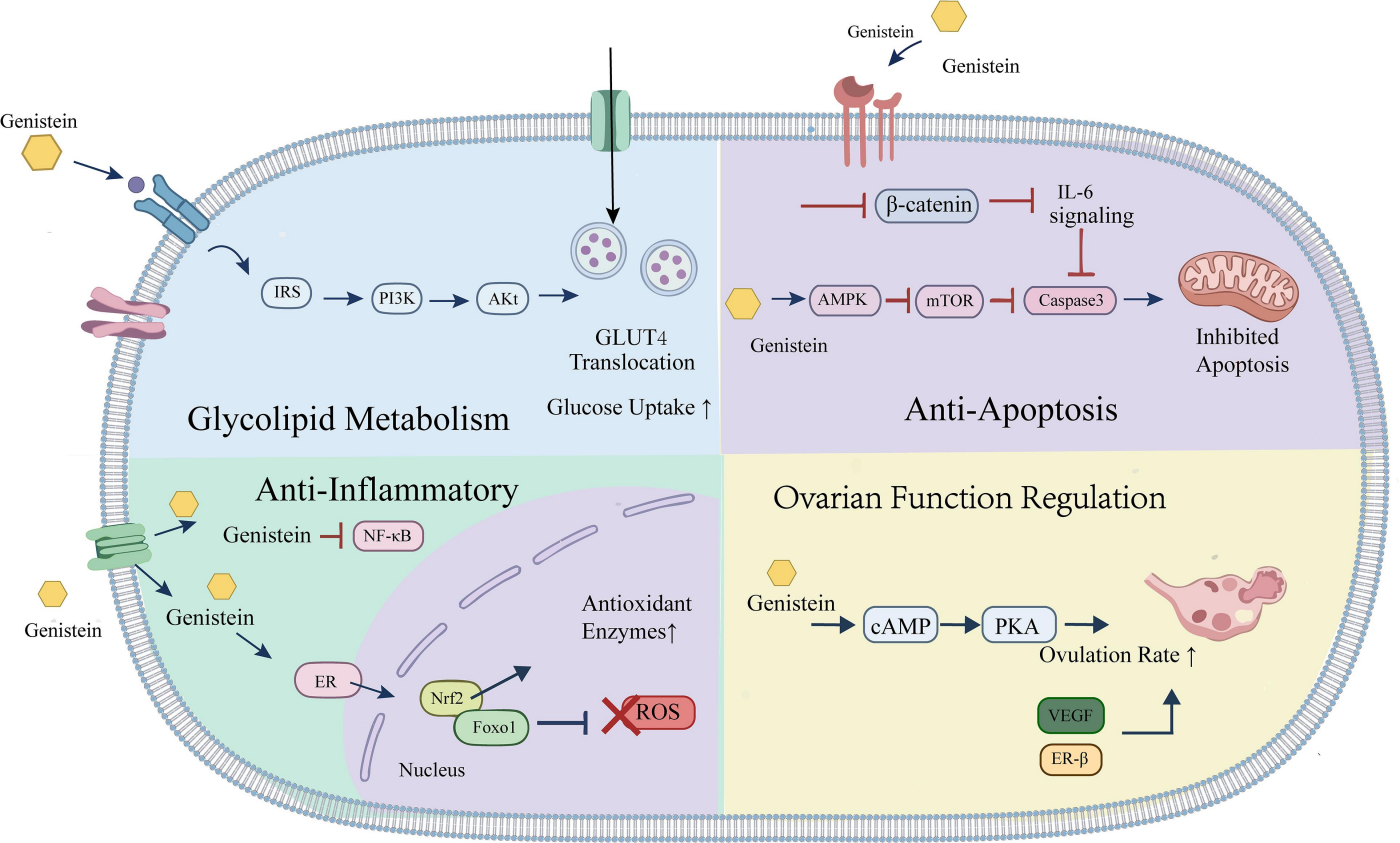
Multi-target regulatory mechanisms of genistein in PCOS.

Given the central role of IR in PCOS, improving insulin sensitivity has become a key therapeutic target. Preclinical evidence strongly supports genistein's efficacy in mitigating IR. In a letrozole-induced PCOS rat model, 42 days of genistein treatment increased GLUT4 translocation to the cell membrane, improved peripheral glucose uptake, and reduced IR, suggesting that genistein helps maintain glucose homeostasis (34). Studies in diabetic mouse models have further provided indirect evidence supporting the potential of genistein to improve insulin resistance. Yang et al. (35) found that an 8-week genistein intervention altered the gut microbiota, particularly by increasing Bacteroides and Prevotella, which was associated with elevated circulating short-chain fatty acids, activation of the AMPK pathway, and enhanced GLUT4 translocation in skeletal muscle. These changes collectively improved hyperglycemia, hyperlipidemia, systemic inflammation, and insulin sensitivity. Similarly, Makena et al. (36) reported that genistein, alone or in combination with bitter melon fruit, reduced total cholesterol, triglycerides, LDL, and VLDL levels. Together, these findings indirectly support the notion that genistein may ameliorate insulin resistance in diabetic mice by modulating gut microbiota and improving metabolic homeostasis (Table 1; Figure 2).

**Table 1:** The regulatory effects of genistein in PCOS.

Mechanis of action	Signaling pathways	Changes in molecules/indicators	Mechanisms summary	References
Improve insulin resistance	AMPK IRS-PI3K-Akt	GLUT4↑; LDL↓; VLDL↓; SCFAs↑; TC↓; TG↓	Modulates gut microbiota → ↑SCFAs → Activates AMPK; Enhances GLUT4 translocation; Protects β-cells.	(25, 34–36)
Glycolipid Metabolism	ERβ; Akt/mTOR; Adiponectin/APPL1; PPAR-α/γ	APPL1↑；HDL-C↑ TC↓; TG↓；SREBP-1c↓; FASN↓	Binds ERβ to inhibit mTOR → ↓Lipogenesis; ↑Adiponectin → Activates PPAR-α for fatty acid oxidation.	(39, 46, 48, 50, 53)
Regulate ovarian granulosa cell reproduction	β-catenin/IL-6;AMPK/mTOR; Caspase-3/PAR	Bcl-2↑; Bax↓; p-AMPK↑; p-mTOR↓; LC3↑ (Autophagy); Caspase-3↓; PARP1↑; T↓	Regulates Bax/Bcl-2 (Anti-apoptosis); Activates autophagy via AMPK/mTOR; Blocks inflammator*y* axis to reduce androgen damage.	(32, 57, 59–61)
Improve ovulation disorders	ER-Nrf2-Foxo1; cAMP/PKA	LH/FSH↓; AMH↓; VEGF↑; ER-β↑;T↓; FOXL-2↑; TGF-β↓; VEGF↑	Repairs mitochondrial function to restore cycles; Activates GPCR-cAMP/PKA to ↑Ovulation; Preserves follicle reserve.	(60, 63–65)
Anti-inflammation	NF*κ*B; PGs; INOS	TNF-α↓, IL-6↓, IL-1β↓；PGE2↓; IL-10↑; TGF-β↑; Macrophage infiltration↓	Prevents NF-κB nuclear translocation → ↓Pro-inflammatory cytokines; ↑Anti-inflammatory cytokines; Reduces macrophage infiltration.	(69, 73, 74, 76)
Antioxidant	Nrf2-Keap1-ARE	Nrf2↑ SOD↑ (SOD1), CAT↑, GSH-Px↑ TAC↑ MDA↓, Protein carbonyls↓	Competitively inhibits Keap1 → Promotes Nrf2 nuclear translocation → Activates antioxidant enzymes; Scavenges ROS, inhibits lipid peroxidation.	(82, 83, 85–88)

↑: Increased expression or activity; ↓: Decreased expression or activity.

However, direct clinical evidence in PCOS populations remains scarce, necessitating careful extrapolation from related metabolic studies. To date, no large-scale randomized controlled trial (RCT) has specifically evaluated genistein's impact on IR in PCOS patients. Current clinical support is primarily derived from studies on obese or diabetic individuals. A clinical study involving 45 participants demonstrated that after obese participants took genistein for 2 months, the composition of the gut microbiota underwent significant changes, along with increased phosphorylation levels of 5'-adenosine monophosphate-activated protein kinase (AMPK) and significantly upregulated expression of genes involved in fatty acid oxidation in skeletal muscle. These changes led to increased levels of β-oxidation, ω-oxidation metabolites, acylcarnitines, and ketone bodies in the circulation, ultimately effectively reducing insulin resistance (37). Additionally, preliminary evidence suggests genistein may promote pancreatic β-cell regeneration, thereby supporting insulin secretion capacity (38). While these findings are promising and mechanistically align with preclinical data, they are limited by small sample sizes and the absence of PCOS-specific cohorts. Therefore, although genistein shows potential as a promising adjunctive therapy for the metabolic and reproductive abnormalities in PCOS, its clinical application requires validation through well-designed, large-scale RCTs focusing on PCOS populations.

### 3.2 Genistein improves glucose and lipid metabolism

Abnormal glucose and lipid metabolism is another major feature of PCOS and is usually characterized by elevated LDL, triglycerides, and total cholesterol, along with reduced HDL-C (39). Obesity, which affects up to 60% of women with PCOS (40), worsens clinical outcomes such as menstrual irregularity, infertility, miscarriage, hyperandrogenism, type 2 diabetes, and metabolic syndrome (41, 42). Mechanistically, visceral adipose tissue contributes to hyperandrogenism through aromatase-mediated estrogen conversion and IR-induced suppression of hepatic SHBG (43, 44). Together, these processes worsen both reproductive and metabolic dysfunction in PCOS.

Preclinical evidence indicates that genistein may improve glucose and lipid metabolism by increasing HDL-C, lowering total cholesterol, and enhancing insulin sensitivity, all of which are key therapeutic targets for the metabolic dysfunction associated with PCOS. From a mechanistic perspective, genistein displays a substantially lower binding affinity for estrogen receptors than endogenous estradiol and exhibits only a relative preference for ERβ. Accordingly, it should be more appropriately classified as a selective estrogen receptor modulator (SERM) rather than a high-affinity ERβ-specific agonist. This SERM-like property enables genistein to modulate ERβ signaling and downstream effectors in a finely tuned manner, thereby exerting favorable effects on lipid metabolism while reducing the potential risks associated with excessive estrogenic exposure (45). Mammalian target of rapamycin (mTOR) is a major regulator of lipogenesis. *In vitro* studies have shown that genistein can inhibit the Akt/mTOR pathway via this SERM-like activity at ERβ, which downregulates the lipogenic transcription factor SREBP-1c and its target gene fatty acid synthase. This suppresses hepatic *de novo* lipogenesis while promoting fatty acid β-oxidation (39). Animal studies support this mechanism. In ovariectomized rats, genistein reduced hepatic triglyceride accumulation in a manner similar to an ERβ agonist, suggesting that it may suppress liver fat buildup modulation of ERβ signaling in estrogen-deficient states (46). Similarly, in diet-induced obese male rats, genistein inhibited hepatic mTORC1 activity and reduced Akt phosphorylation (47). While preclinical data are compelling, there remains a significant paucity of human evidence to support its clinical application in PCOS, owing to the lack of dedicated cohort studies tailored to its unique pathological features. A meta-analysis found that genistein effectively improves glucose and lipid metabolism by increasing HDL-C levels, lowering total cholesterol levels, and enhancing insulin sensitivity (48). However, existing meta-analyses suffer from considerable heterogeneity in study populations, often encompassing postmenopausal women, diabetic patients, and other diverse groups, thereby limiting the specificity of their conclusions to the unique pathophysiological characteristics of PCOS.

Adiponectin is an anti-inflammatory adipokine that improves insulin sensitivity and reduces hyperglycemia, atherosclerosis, and inflammation (49). Genistein significantly increases adiponectin levels in both serum and tissues. In PCOS-IR model rats, a 3-week genistein intervention increased serum adiponectin and APPL1 by 69% and 37%, respectively, suggesting that genistein may improve IR and glucose metabolism through adiponectin- and retinol-binding protein 4 (RBP4)-related pathways (50). Genistein increased estrogen receptor β (ERβ), forkhead box O1, nicotinamide phosphoribosyl transferase, sirtuin1 (SIRT1), phospho (p)-adenosine 5'-monophosphate-activated protein kinase (AMPK), peroxisome proliferator-activated receptor γ coactivator-1α (PGC-1α), p-ACC, and CPT-I protein levels, whereas the SREBP-1c and FAS levels were decreased (51). PPAR-γ is also a key regulator of lipid metabolism and influences triglyceride synthesis, lipid uptake, and cholesterol efflux (52). In models of fatty liver disease, genistein's effects on hepatic fat and oxidative stress have been linked to increased adiponectin and reduced PPAR-γ expression (53). Higher adiponectin may also support the reproductive axis by influencing GnRH and LH secretion (54) (Table 1).

Beyond hepatic signaling, genistein also acts directly on adipose tissue. In ovariectomized rat models, it increased the activity of enzymes involved in fatty acid oxidation, such as succinate dehydrogenase and carnitine palmitoyltransferase, which accelerated β-oxidation and reduced adipocyte size. By promoting lipolysis and fat oxidation, genistein may help reduce central obesity and improve IR associated with elevated free fatty acids (55). It also reduces hepatic triglyceride accumulation and steatosis caused by high-fat or high-carbohydrate diets (56) (Figure 2). Taken together, these findings suggest that genistein may regulate glucose and lipid metabolism mainly by increasing adiponectin and modulating adipokine signaling. It may therefore serve as a novel intervention for PCOS-related metabolic disturbances.

## 4 Therapeutic effects of genistein on ovarian function in PCOS

### 4.1 Genistein regulates proliferation, apoptosis, and autophagy in granulosa cells

Ovarian granulosa cells are essential for follicular function, providing nutritional support and mediating steroidogenesis (57). In PCOS, dysregulated autophagy, apoptosis, and steroidogenesis in these cells contribute to follicular arrest and ovulatory dysfunction (58). Evidence suggests that genistein has a dose-dependent biphasic effect on granulosa cells. At lower concentrations (approximately 10–25 μM), it promotes cell proliferation and improves cellular activity. At higher concentrations (50–100 μM and above), it inhibits cell growth and alters steroid hormone production, usually decreasing 17β-estradiol and increasing progesterone levels (59). These findings suggest that genistein may regulate ovarian function by modulating genes involved in steroid synthesis in a dose-dependent manner.

Genistein also protects granulosa cell viability by regulating apoptosis. Under stress conditions such as radiation or serum deprivation, it exhibits anti-apoptotic activity. Mechanistically, genistein upregulates the anti-apoptotic protein Bcl-2 and downregulates Bax, thereby reducing the Bax/Bcl-2 ratio. This inhibits cytochrome c release and caspase-3 activation (60). In an IR-induced hyperandrogenism model using human ovarian granulosa cells and a mouse PCOS model, genistein, particularly in combination with secreted frizzled-related protein 4 (SFRP4), reduced intraovarian testosterone levels and was associated with improvements in PCOS-related features. This effect was partly mediated by regulation of the nuclear β-catenin/IL-6 signaling axis (61). These findings suggest that genistein may help maintain granulosa cell survival and reduce follicular atresia via its anti-apoptotic effects. Its potential regulatory role in autophagy, the mechanisms of which are elaborated in the following section, may represent an additional contributor to its ovarian protective actions (Figure 2).

Additional evidence comes from serum-deprived porcine granulosa cells, where genistein promoted AMPK phosphorylation and inhibited mTOR phosphorylation, leading to LC3 activation and autophagy induction. At the same time, it inhibited caspase-3 cleavage and PARP1 activation, which reduced apoptosis (59). These findings indicate that genistein maintains GC survival via synergistic regulation of autophagy activation and apoptosis inhibition, relying on dual modulation of the AMPK/mTOR and caspase-3/PARP1 pathways, representing a potential strategy to prevent follicular atresia. Despite robust mechanistic data from *in vitro* and animal models, direct clinical evidence supporting genistein's effects on GC function in PCOS patients is currently lacking. Crucially, the clinical translation of these findings is hindered by the paucity of PCOS-specific trials. Although evidence extrapolated from general fertility studies suggests a potential benefit for oocyte quality, the findings remain inconsistent and lack direct *in vivo* validation of granulosa cell biomarkers. Moreover, the biphasic effects observed in preclinical studies raise important concerns regarding dosage. Achieving the supraphysiological concentrations required for therapeutic efficacy through standard supplementation is likely impractical and may pose safety risks, thereby complicating the transition to clinical application (Table 1).

In summary, while preclinical studies demonstrate that genistein can modulate the ovarian microenvironment by regulating steroidogenesis, autophagy, and apoptosis in GCs *in vitro* and in animal models, caution is warranted regarding its clinical translation. The current evidence base, derived from heterogeneous experimental systems, primarily delineates mechanistic interactions rather than established clinical outcomes. Future investigations should prioritize well-designed clinical trials that move beyond surrogate metabolic markers to directly evaluate effects on follicular dynamics and oocyte quality in PCOS patients. Such trials are essential to define safe and efficacious dosing regimens that account for the compound's complex, dose-dependent bidirectional effects, thereby bridging the gap between promising mechanistic observations and evidence-based therapeutic applications.

### 4.2 Genistein ameliorates ovulatory dysfunction

PCOS is commonly associated with oligo-ovulation or anovulation. Evidence suggests that genistein can act directly on the ovary to support follicular development and improve ovulatory function. In a randomized animal study, Khezri et al. (62) showed that a 14-day genistein intervention increased gonadotropin and steroid hormone production, reduced follicular atresia, and improved follicular morphology in PCOS rats. These results suggest that genistein may benefit ovarian tissue by regulating the reproductive endocrine axis and maintaining follicular microenvironment stability.

Further studies in PCOS mouse models indicate that genistein improves ovarian antioxidant defense and mitochondrial function, mainly through ER-Nrf2-Foxo1 signaling axis (63). This mechanism restored estrous cyclicity in PCOS mice and concomitantly reduced circulating levels of testosterone, AMH, and LH, while normalizing the disrupted LH/FSH ratio toward physiological ranges and decreasing the number of cystic follicles. These findings underscore the therapeutic potential of genistein in managing PCOS. Genistein may help normalize sex hormone profiles and restore regular ovulation in affected individuals.In a randomized, controlled animal study using a PCOS rat model, genistein treatment was associated with more regular estrous cycles and significantly reduced final body weight and weight gain. Histological analysis showed increased follicle counts, along with endometrial epithelial hyperplasia and stromal edema (64). The study linked these effects were mediated by genistein's regulation of ERα, ERβ, and VEGF. However, whether these histological changes translate to improved fertility in humans remains to be verified.

At the molecular level, genistein may reverse ovarian apoptosis by increasing ER-β and FOXL-2 and decreasing TGF-β expression. This may help prevent excessive activation of primordial follicles into the growing pool (60), This suggests an integrated mechanism involving antioxidant, anti-apoptotic, and signaling effects that protect the ovary from stress damage. In this way, genistein may preserve the primordial follicle reserve, support the growing follicle pool, and reduce follicular atresia, thereby improving ovulatory function in PCOS. Moreover, computational and animal studies suggest that genistein binds with high affinity and stability to ovarian G protein-coupled receptors, in some models even more strongly than estradiol (65). This binding activates the cAMP/PKA signaling cascade, which may increase ovulation rates and improve fertility by helping synchronize estrous cycles. This points to the cAMP/PKA pathway as another possible mechanism behind genistein's reproductive benefits (Figure 2; Table 1).

Overall, preclinical evidence suggests that genistein may ameliorates PCOS-associated ovulatory dysfunction by restoring estrous cyclicity and modulating follicular dynamics. However, direct clinical validation remains elusive. The absence of large-scale RCTs confirming improvements in human ovulation or live birth rates, coupled with pharmacokinetic barriers that preclude the safe replication of efficacious preclinical concentrations via oral supplementation, necessitates a cautious interpretation of its reproductive benefits.

## 5 Genistein attenuates inflammation and oxidative stress in PCOS

### 5.1 Genistein suppresses proinflammatory cytokine expression

Chronic low-grade inflammation is an important contributor to PCOS development (66). Clinical epidemiological data indicate that PCOS patients exhibit characteristic aberrations in their serum inflammatory mediator profile, specifically marked by significantly elevated levels of C-reactive protein (CRP), tumor necrosis factor-alpha (TNF-α), interleukin-6 (IL-6), and IL-18 (67). While these cytokines are deeply implicated in the pathophysiological processes of PCOS, their vital regulatory roles in maintaining ovarian homeostasis within physiological concentration ranges warrant emphasis. For instance, IL-6 participates in immune responses by modulating T lymphocyte activation and B lymphocyte differentiation. Conversely, TNF-α demonstrates classic concentration-dependent bidirectional regulatory effects: at physiological levels, it mediates theca cell proliferation and coordinately regulates follicular development and corpus luteum formation (68). However, within the characteristic chronic inflammatory milieu of PCOS, the dysregulated expression of these cytokines and the sustained hyperactivation of their signaling pathways disrupt the intricate local immune-endocrine regulatory network of the ovary. Animal studies that the specific blockade of excessive TNF-α and IL-6 signaling effectively ameliorates ovulatory dysfunction and rescues luteal defects. This finding mechanistically elucidates the paradigm shift from physiological signaling to pathological chronic inflammation as a critical driver of PCOS phenotype manifestation (69).

Nuclear factor-kappa B (NF-κB) is a central pathway linking inflammation to PCOS. NF-κB is a family of transcription factors that regulates immune and inflammatory responses, cell growth, and survival (70). In PCOS, NF-κB is overactivated, promoting excessive production of proinflammatory mediators and chronic ovarian inflammation. Genistein exerts its anti-inflammatory effects primarily by inhibiting NF-κB activation and nuclear translocation, thereby reducing TNF-α, IL-6, IL-1β, and PGE2 expression (69, 71, 72). It may also increase anti-inflammatory cytokines such as IL-10 and TGF-β (73) (Table 1). In animal models, genistein reduced macrophage infiltration and attenuated hepatic inflammation, and its effects may be enhanced when combined with metformin (74). A meta-analysis of 51 included RCTs with subgroup analysis demonstrated that long-term intervention with low-dose genistein significantly reduces interleukin-6 (IL-6) and tumor necrosis factor-α (TNF-α) levels (75). However, because these trials primarily included postmenopausal women or individuals with metabolic syndrome, the reported efficacy and safety outcomes may not be directly applicable to the PCOS population, which is characterized by a distinct hormonal environment and body composition. Notably, genistein's anti-inflammatory effect appears to be dose-dependent and may work primarily additive rather than formally synergistic effects. In mouse models, a dose of 0.3 mmol/kg improved liver fibrosis and cell death by lowering pro-inflammatory and pro-fibrotic cytokines, as well as caspase-3 (76). These findings suggest that optimized dosing and combination therapy may improve metabolic and inflammatory outcomes. NF-κB appears to be a primary target of genistein. While NF-κB activation can trigger downstream kinase pathways such as PI3K/Akt, and MAPKs including p38, ERK1/2, and JNK (77), genistein counteracts this by inhibiting NF-κB nuclear translocation and downstream cytokine production. Beyond NF-κB, genistein also modulates other inflammatory mediators, including prostaglandins, inducible nitric oxide synthase, and reactive oxygen species, giving it a broad anti-inflammatory profile (69) (Figure 2).

In summary, while genistein alleviates chronic inflammation in preclinical PCOS models by suppressing NF-κB signaling and limiting immune cell infiltration, its clinical utility remains at the hypothetical stage. Future randomized controlled trials should focus on PCOS-specific cohorts to validate these anti-inflammatory effects and establish optimal dosing strategies that account for the interplay among inflammation, insulin resistance, and reproductive function.

### 5.2 Genistein reduces oxidative stress

Oxidative stress, defined as an imbalance between reactive oxygen species (ROS) production and antioxidant defense, is a major component of PCOS pathophysiology (78). It interacts with insulin resistance, inflammation, and ovarian dysfunction, creating a vicious cycle. The Keap1-Nrf2-ARE signaling pathway plays a central role in cellular defense against oxidative stress (79). Under normal conditions, Nrf2 is bound to Keap1 and degraded. Under oxidative stress, Nrf2 dissociates from Keap1, translocates to the nucleus, and activates transcription of antioxidant enzymes such as superoxide dismutase, catalase, and glutathione peroxidase (80, 81).

Genistein has strong antioxidant properties and can reduce oxidative damage through multiple mechanisms, including modulation of intracellular signaling and enhancement of free radical scavenging capacity (82, 83). In a randomized controlled study of 120 PCOS rats, genistein reduced LH, FSH, testosterone, and oxidative stress markers while increasing antioxidant enzyme activity and improving ovarian histology (84). It also activates the Nrf2 pathway by modifying Keap1, thereby promoting Nrf2 nuclear translocation and upregulating downstream antioxidant genes. In mouse ovarian toxicology models, genistein activated the Nrf2-Keap1 pathway, increased catalase, glutathione peroxidase, and total SOD activity, and reduced malondialdehyde (MDA) accumulation (85). Additional studies have shown that genistein increases Nrf2, SOD1, and CAT expression and decreases MDA and protein carbonyl levels, further supporting its antioxidant role (86, 87). In a clinical study of postmenopausal women, genistein supplementation improved metabolic parameters and enhanced total antioxidant capacity. However, direct clinical evidence in PCOS patients remains limited (88). However, the distinct pathophysiological environment of PCOS, characterized by hyperandrogenism and insulin resistance, limits the translational relevance of these findings derived from postmenopausal populations (Table 1).

In summary, genistein exerts a multifaceted antioxidant effect by activating the Nrf2-Keap1 axis and enhancing endogenous enzymatic activity (Figure 2). However, its progression to a clinical therapeutic agent requires validation. Future RCTs should focus on PCOS-specific cohorts to confirm whether systemic Nrf2 activation translates to measurable improvements in ovarian function and redox balance.

## 6 Limitations

Despite a robust body of preclinical evidence suggesting that genistein ameliorates insulin resistance, dyslipidemia, and the ovarian microenvironment, a significant translational hiatus persists, preventing its endorsement by regulatory agencies such as the FDA for clinical PCOS management. This gap stems primarily from suboptimal pharmacokinetics, complex dose-dependent biphasic effects, and a critical dearth of large-scale RCTs tailored to PCOS heterogeneity.

First, pharmacokinetic limitations and the animal-to-human translational gap constrain clinical extrapolation. The clinical development of genistein is hindered by several pharmacokinetic drawbacks, including poor water solubility, rapid systemic metabolism, and low oral bioavailability (89). Critically, the bioactivity of genistein is tightly contingent on metabolic activation orchestrated by the gut microbiota. Plant-derived genistein exists primarily as biologically inert glycosides, requiring hydrolysis by bacterial β-glucosidases to yield absorbable aglycones. Subsequent biotransformation—notably the production of the potent metabolite equol—is exclusively governed by the host's gut microbial composition (90). Given substantial interindividual variability in microbiota, only a subset of the population harbors equol-producing taxa. This metabolic heterogeneity directly precipitates variable plasma concentrations and therapeutic responses, likely driving the poor reproducibility observed across clinical trials (91). More importantly, most mechanistic data are derived from animal studies. However, clear species-specific and sex-dependent differences exist between animals and humans in terms of drug absorption, metabolism, and endocrine regulation. For example, the absorption rate of genistein and its metabolites is nearly 100% in female rats but only approximately 56% in male rats, and absorption efficiency also differs across intestinal regions (92). Since the molecular basis of these differences is unresolved, direct extrapolation of animal “effective doses” to PCOS patients introduces significant uncertainty.

Second, genistein exhibits dose-dependent bidirectional effects, and its therapeutic window remains undefined. Genistein exerts a classic biphasic effect on female health. At low doses, it acts as a weak estrogen agonist and may promote lipoprotein lipase (LPL) activity through activation of PPARγ and downstream signaling, thereby facilitating lipid metabolism. In contrast, at high or suprapharmacological doses, genistein may exert anti-estrogenic effects, markedly suppress LPL and other key metabolic enzymes, and potentially produce opposite metabolic outcomes (93, 94). Clinical pharmacokinetic studies show that the plasma concentration–time relationship of orally administered genistein remains approximately linear only at doses below 150 mg, suggesting that intestinal absorption may become saturated at higher doses. Although doses below 300 mg are generally well tolerated in healthy individuals (95). Notably, the classification of genistein as a potential “endocrine disruptor” in prior literature (18) derives almost exclusively from *in vitro* assays and high-dose animal models. To date, no clinical evidence supports the applicability of this classification to humans under physiological exposure levels; consequently, its translational relevance to human health remains limited. Animal studies further support this concern. Jefferson et al. reported that developmental exposure to genistein induced adverse reproductive effects in mice, including progressive deterioration of ovarian function and estrous cyclicity. In mouse models, exposure to 0.5, 5, or 25 mg/kg genistein reduced fertility, whereas 50 mg/kg caused infertility. Further work suggested that long-term exposure to 25 mg/kg genistein may increase the risk of uterine adenocarcinoma later in life (96). In addition, studies in 3T3-L1 adipocytes showed that low-dose genistein (10 nM) mimicked estradiol, upregulated Slc2a4/GLUT4 expression, and enhanced glucose uptake, whereas higher doses significantly suppressed expression, suggesting a shift from pro-metabolic effects toward potential disruption of glucose metabolism (97). Notably, high-quality pharmacokinetic data in PCOS populations remain scarce, representing a key barrier to defining a precision therapeutic window. Collectively, these findings indicate that the dose-dependent effects of genistein are complex and bidirectional, underscoring the need to define a precise therapeutic window that improves core PCOS pathology without inducing systemic adverse effects. At present, the EU/Polish dietary supplement guideline supports a daily genistein dose of 50 mg; however, this recommendation reflects a conservative safety threshold rather than an optimal therapeutic dose for PCOS treatment (98) (Table 2).

**Table 2:** Summary of dose-dependent bidirectional effects of genistein.

Model system	Exposure condition/dose	Primary beneficial outcomes	Primary adverse/risk outcomes	References
Cellular Studies (Granulosa Cells/Adipocytes)	Low Dose 10–25µM (GCs) 10 nM (Adipocytes)	①Promotes granulosa cell proliferation and viability. ②Mimics estradiol, upregulates GLUT4 expression, and enhances glucose uptake.	No overt cytotoxicity observed.	(97)
	High Dose 50–100 µM (GCs) > 10 nM (Adipocytes)	—	①Inhibits granulosa cell growth. ②Disrupts steroid hormone profile (↓E2, ↑P4). ③Suppresses GLUT4 expression and impairs glucose uptake.	(97)
Animal Models (Reproductive/Developmental)	High Dose 0.5–50 mg/kg	—	①Reproductive Toxicity: Impairs ovarian function and estrous cyclicity upon developmental exposure. ②Infertility: Reduced fertility at 25 mg/kg; complete infertility at 50 mg/kg. ③Carcinogenic Risk: Long-term exposure (25 mg/kg) increases the risk of uterine adenocarcinoma later in life.	(96)
Molecular Mechanisms (Metabolic Pathways)	Low Exposure (Pharmacological Levels)	①Pro-metabolic: Activates PPARγ and downstream targets, promotes LPL activity, and improves lipid metabolism.	—	(93, 94)
	High Exposure (Suprapharmacological Levels)	—	①Metabolic Suppression: Exhibits anti-estrogenic effects, significantly inhibits key metabolic enzymes (e.g., LPL), leading to metabolic outcomes opposite to those seen at low doses.	(93, 94)
Human Studies (PK/Endocrine)	≤150 mg/day (Linear PK Range)	①Safety: Well tolerated in healthy subjects. ②PK Profile: Plasma concentration-time curve is approximately linear, indicating stable absorption.	—	(95)
	>150 mg/day (Saturation Range)	—	①PK Risk: Intestinal absorption approaches saturation; nonlinear PK increases inter-individual variability in exposure. ②Endocrine Disruption: Binds ERα/ERβ, interferes with testosterone biosynthesis (↓Testosterone by 19% in men), and impairs testicular function.	(18, 95, 108)

This table summarizes evidence across biological scales using system-specific units (cellular: µM/nM; animal: mg/kg; human: mg/day). Values across scales are not linearly convertible and must be interpreted considering bioavailability; “—” indicates that no corresponding specific effect was observed in the existing literature under that exposure condition.

Third, the level of clinical evidence remains low and hard endpoint data are lacking. Clinical research on genistein in women with PCOS remains exploratory and is limited by the absence of large-scale, multicenter RCTs. Existing studies are generally small and short term, with most focusing on surrogate endpoints rather than clinically meaningful outcomes. These surrogate measures include fasting glucose, HbA1c, triglycerides, MDA/TAC, and sex hormone profiles. However, robust data on hard endpoints that are directly relevant to patients—such as ovulation recovery, menstrual regularity, spontaneous pregnancy, and live birth rates—are still lacking. In addition, no well-established stratification strategies exist for different PCOS phenotypes (e.g., obese vs. non-obese, metabolically driven vs. hyperandrogenism-driven), nor has an optimal treatment duration been defined.

Finally, long-term safety remains an important unresolved issue, especially in patients with endocrine comorbidities. The effects of genistein on thyroid function remain controversial. Although meta-analyses suggest that soy intake does not exert a significant clinical impact on thyroid function in healthy adults (99), phytoestrogen supplementation may increase the risk of progression to overt hypothyroidism in patients with subclinical hypothyroidism (100, 101). Further studies are needed to clarify the appropriate dosage and target population. In addition, soy-derived metabolites such as genistein may trigger allergic reactions. Given the current lack of high-quality evidence, the efficacy of genistein in patients with severe PCOS appears limited. Moreover, as a phytoestrogen, genistein has relatively mild biological activity, and current evidence does not support its use as a stand-alone first-line therapy for PCOS. If future high-quality studies confirm its clinical value, genistein will more likely be positioned as an adjunctive or combination therapy rather than a primary treatment option (87).

## 7 Future perspectives

Genistein, as a potential adjunctive therapeutic candidate for PCOS, has shown promise in improving metabolic homeostasis, regulating sex hormones, and restoring ovarian function. To overcome its limitations, researchers have been developing novel delivery systems to enhance genistein's solubility, stability, and targeting efficiency. A variety of drug delivery platforms have been designed to protect and stabilize genistein and address the challenge of its low bioavailability (102). Previous studies have shown that drug-loaded nanoparticles, solid lipid nanoparticles (SLNs), and nanostructured lipid carriers have been explored as potential therapeutic approaches for multiple diseases (103, 104). These systems can significantly improve genistein's solubility, stability, and absorption, thereby increasing oral bioavailability and potentially enhancing therapeutic responses by improving tissue permeability (18, 20). In addition, genistein formulations may be further optimized to improve routes of administration, including tablet, microparticle, and micellar systems (105). With these advances, low bioavailability may no longer represent a major barrier to clinical translation.

Given that genistein is a relatively mild phytoestrogen and that monotherapy may offer limited efficacy in patients with severe PCOS, future research should focus on evaluating its potential as an adjunctive treatment for PCOS and investigating its effects across different PCOS phenotypes. Studies should also examine the synergistic effects of genistein with conventional therapies, identify the optimal therapeutic dose, and minimize adverse effects. Combination therapy has shown encouraging synergistic potential. For example, co-administration with metformin may further improve insulin sensitivity and reduce gastrointestinal adverse effects associated with metformin (106). When combined with traditional ovulation-inducing agents, genistein may help optimize the ovarian microenvironment and improve follicular quality and ovulation success. Likewise, in combination with dietary supplements such as quercetin, it may exert complementary effects on lipid reduction and immune modulation (107).

To facilitate the clinical translation of genistein, large-scale, multicenter, randomized controlled trials are needed. Future studies should focus on: (1) optimizing pharmacokinetic properties through novel delivery systems; (2) defining the optimal therapeutic window using individualized dosing strategies; and (3) systematically evaluating the synergistic efficacy and long-term safety of combination therapies. Such studies will help clarify the real-world clinical value of genistein regarding dose optimization, administration regimens, combination strategies, and long-term safety.

## 8 Conclusion

**Figure 3:**
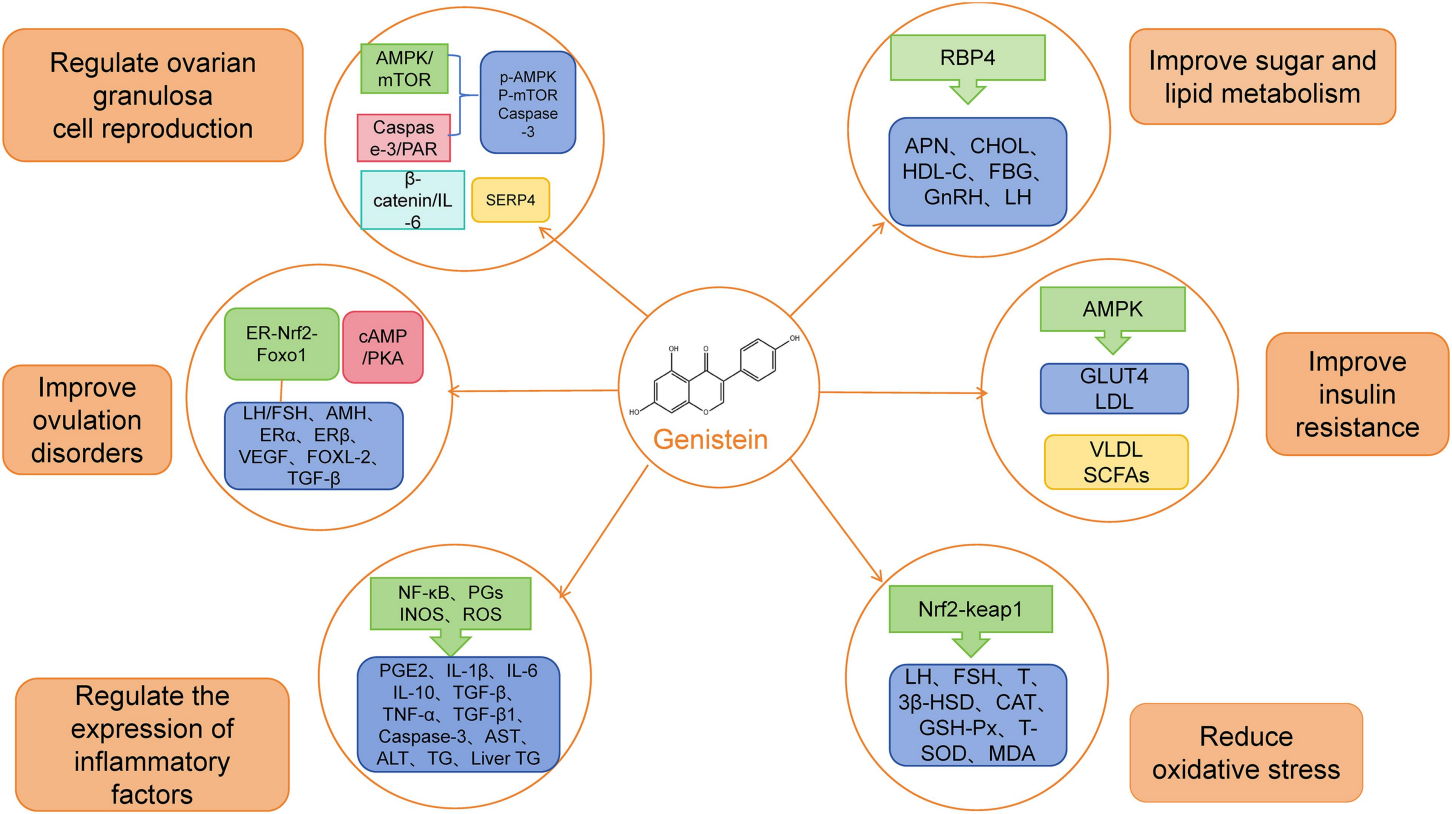
Therapeutic mechanisms of genistein in PCOS.

Genistein is a natural isoflavone with promising multi-target activity predominantly demonstrated in preclinical models of PCOS. *In vitro* and animal studies suggest it may improve insulin resistance and lipid metabolism through AMPK-, mTOR-, and adiponectin-related pathways; alleviate reproductive dysfunction by modulating granulosa cell survival, hormonal balance, and follicular development; and mitigate inflammation and oxidative stress via NF-κB suppression and Nrf2 activation (Figure 3). However, direct clinical translation of these findings remains uncertain, as high-quality human data in PCOS populations are currently limited. Although clinical application is currently restricted by low bioavailability and dose-dependent biphasic effects, genistein remains a promising adjunct candidate for multidimensional PCOS management pending further validation. Therefore, rigorous clinical trials are warranted to confirm its efficacy, optimize pharmaceutical delivery, and establish safe and effective dosing strategies tailored to PCOS pathophysiology.
